# Drought tolerant wheat IND-ØØ412-7 is nutritionally equivalent to its Non-Transgenic Comparator

**DOI:** 10.1080/21645698.2022.2079179

**Published:** 2022-06-03

**Authors:** Patricia V. Miranda, Bernardo F. Iglesias, María V. Charriere, Moisés Burachik

**Affiliations:** a Instituto de Agrobiotecnología Rosario (INDEAR), Santa Fe, Argentina; b Consejo Nacional de Investigaciones Científicas y Técnicas (CONICET), Argentina; c Instituto Nacional de Tecnología Agropecuaria (INTA) - Estación Experimental Agropecuaria Pergamino, Buenos Aires, Argentina

**Keywords:** Broiler feeding, drought-tolerant wheat, GM wheat, HB4 wheat, transgenic wheat

## Abstract

Expression of the HAHB4 sunflower transcription factor confers drought tolerance to wheat event IND-ØØ412-7 (HB4® wheat). After confirming the compositional equivalence of event IND-ØØ412-7 with conventional wheat, its nutritional similarity to its non-genetically modified (GM) counterpart was analyzed by performing a 42-day broiler feeding study. Isoenergetic diets containing 40% flour from wheat event IND-ØØ412-7, its non-GM counterpart Cadenza, and a commercial variety were included in the study. Broilers’ performance was analyzed by measuring feed intake, weight gain, feed conversion, and time to reach 2.8 kgs. The yield was evaluated by carcass weight, breast meat, and abdominal fat. No differences were found between wheat event IND-ØØ412-7 and the non-GM counterpart. A few significant differences were found with the commercial variety which were associated with the genetic background, different from the other two materials. These results support the nutritional equivalence of event IND-ØØ412-7 with conventional wheat.

## Introduction

Wheat is the staple food for 35% of the world’s population.^1,2^ This cereal is not only a primary source of carbohydrate energy but also provides other important nutrients. Except for the limiting content of one essential amino acid (lysine), the levels of proteins, fiber, and minor components including lipids, vitamins, minerals, and phytochemicals (phenolics and terpenoids) all make wheat a significant contribution to a wholesome diet.^3^

To meet the demand of a growing human population estimated at more than 9.7 billion by 2050, wheat production will need to increase current values by 60% to 110%.^4,5^ Drought is the most important environmental stress that limits crop productivity. Wheat event IND-ØØ412-7 was developed to achieve water-restriction tolerance by the introduction of the *HaHB4* sunflower gene encoding the transcription factor HAHB4.^6^ This protein is known to be involved in the regulation of developmental processes associated with the response of plants to environmental stress.^7,8^ This wheat event also contains the *bar* gene from *Streptomyces hygroscopicus* providing the glufosinate-tolerance phenotype.^9–11^

The food safety of wheat event IND-ØØ412-7 could be anticipated by the history of safe use of the source of the introduced gene (sunflower), its mode of action (a transcriptional regulator of normal endogenous pathways, with no novel proteins or metabolites being produced),^12^ and the extremely low levels of HAHB4 expression. These safety features have been validated through a comprehensive set of studies by which regulatory agencies confirmed the safety of this GM wheat.^13,14^

The compositional equivalence of wheat event IND-ØØ412-7 to its non-GM counterpart Cadenza has been recently demonstrated.^15^ Many studies on the safety of food and feed derived from GM plants with improved agronomic traits have shown that once compositional equivalence has been established, no biologically relevant differences in the food/feed nutritional performance should be expected between the GM crop and its near-isogenic counterpart.^16,17^ However, nutritional performance data (e.g., chickens’ growth rate and related endpoints) were often required by regulatory authorities.

Accordingly, the objective of the present work was to confirm the nutritional quality of GM wheat. Here we report the nutritional assessment of wheat event IND-ØØ412-7 through a 42-day feeding assessment of broiler performance and carcass characteristics. Results of this study support the nutritional equivalence of wheat event IND-ØØ412-7 to non-GM wheat.

## Materials and methods

### Samples

The three materials used for the study included mature grain from event IND-ØØ412-7 (HB4), its non-GM counterpart near-isogenic line Cadenza, and a commercial local reference variety (BioINTA 3005). Experimental materials were harvested in the 2013 season at Balcarce (38° S, 58°W), Buenos Aires, Argentina (Permit number 345854–1/12). This location has a clay loam typic argiudoll soil with the following topsoil characteristics: 5.2% of organic matter; pH = 5.6; electric conductivity = 0.1 dS/m. The crop was fertilized according to farm prescription, with Nitrogen and Phosphorus at rates of 156 and 83 kg/ha respectively. Rainfall totalized 452 mm during the crop cycle (June-December), 70 mm below the historic record for the same period (522 mm for average 2000–2018). The mean temperature during the growth period was 12.7°C, and minimum and maximum temperatures resulted favorable for grain production, ranging from 11 to 29°C during anthesis and grain filling. The three genotypes were grown in parallel plots located within the same field and managed with the same usual agronomic practices. Grain samples were checked for mycotoxins presence by chromatography^18^ to confirm that their levels were below the accepted limits for poultry production (Aletheias Laboratory, Ituzaingó, Buenos Aires, Argentina).

### Grain compositional analysis

Moisture, crude protein, ether extract, crude fiber, ash, and total amino acids contents were estimated using near-infrared reflectance (NIR) spectroscopy, a strategy regularly used to assess the composition of plant tissues for feed formulation in commercial broiler production.^19^ The equipment used was a FOSS® 5000 (Foss Analytical A/S, Hillerød, Denmark) with wheatc7 calibration curve for amino acids and pgwheac1_foss calibration curve for the rest of the estimations.

### True metabolizable energy

The true metabolizable energy (TME) of each material, as well as the endogenous losses, were determined by the total collection technique^20^ using cockerels. Briefly, 28 cockerels (Hy-Line W-36) were fasted for 24 hours to clear the gastrointestinal tract. Then, birds were tube-fed 40 g of ground grain from the different wheat genotypes included in the study and placed in metabolic cages with water access only. Each material included in the study, as well endogenous losses, were replicated seven times using one animal per replicate. Excreta collected for 48 hours were dried at 60°C in a forced-air oven and weighed. The gross energy (GE) content of the feed and the excreta were determined in a PARR isoperibolic oxygen bomb calorimeter model 1261 (Parr Instrument Company, Moline, IL, USA) in accordance with the standard method ASTM D2015-85,^21^ and TME was calculated as follows:

$$\begin{array}{rl} & TME\left(kcal/kg\right)=\left(GEintake-GEexc+GEend\right)\\ & \;\;\;\;\;\;\;\;\;\;\;\;\;\;\;\;\;\;\;\;\;\;\;\;\;\;\;\;\;\;\;\;\;\;\;\;\;\;\;\;\;\;\;/\left(Feedintake\right), \end{array}$$

where GEintake: gross energy intake, kcal; GEexc: gross energy excreted by the fed birds, kcal; GEend: gross energy excreted by fasted birds (endogenous losses), kcal; Feed intake, kg.

TME was expressed as kcal/kg and Energy utilization was calculated as a percentage of GE, (i.e., the TME/GE ratio).

### Animals and housing

This study was conducted in accordance with the principles and guidelines for the care and use of experimentation animals.^22^ A total of 360 one-day-old male broiler chicks (Cobb-500) were obtained from a commercial hatchery (Malacate Hatchery, Granja Tres Arroyos, Buenos Aires Province, Argentina). Chickens were vaccinated at hatching for Marek’s and infectious bursal diseases. The chickens were weighed and grouped (15 birds per group) such that all groups had similar mean weight and the weight variation within each group was minimized. Groups were randomly assigned to one of the three treatments and allocated in clean floor pens (1.5 m × 1 m), at a density of 10 birds per sq. meter, with wood shavings as litter. The lighting (compact fluorescent light bulbs) cycle was 16 h of light and 8 h of dark. Room temperature was set at 32°C on day 1, and then gradually decreased to a target room temperature of 23°C on day 28 and kept constant thereafter. Water and feed were provided *ad-libitum* via automatic nipple drinkers and tube feeders.

### Experimental design

A randomized complete block design was used with the three treatments: the transgenic event being tested (IND-ØØ412-7), its non-GM counterpart (Cadenza), and a commercial variety (BioINTA 3005). Blocks were arranged to avoid house environmental variations. Each dietary treatment was offered to 8 pens (one pen per block) of 15 birds each (120 birds/treatment). The pen was considered the experimental unit.

### Experimental diets

Diets were formulated in a three phases program according to the requirements of the chicken´s line^23^ based on wheat, corn, soybean meal, extruded soybean, meat and bone meal, and soybean oil. Materials of the three wheat genotypes were ground upon arrival at the INTA facilities. Experimental diets containing 40% of ground wheat were formulated to meet the same level of TME and the principal amino acids (lysine, methionine+cystine, and threonine) (Table 1). No exogenous enzymes were added to the diets. All diets were supplied in a mash form.

**Table 1.** Diets formulation and composition

		Starter (Days 1 to 14)			Grower (Days 15 to 28)			Finisher (Days 29 to 42)		
		IND^a^	Cadenza^2^	BioINTA^3^	IND^a^	Cadenza^2^	BioINTA^3^	IND^a^	Cadenza^2^	BioINTA^3^
Ingredients (%)										
Corn		21.75	21.52	20.07	22.14	22.36	20.69	22.12	21.94	20.50
Wheat		40.00	40.00	40.00	40.00	40.00	40.00	40.00	40.00	40.00
Extruded soybean		-	-	-	15.00	15.00	15.00	20.00	20.00	20.00
Soybean oil		2.24	2.35	3.52	0.66	0.56	1.84	0.80	0.91	2.08
Soybean meal (45% CP^4^)		28.95	28.99	29.27	15.80	15.76	16.08	11.66	11.69	11.97
Crustacean shells		0.44	0.44	0.43	0.42	0.42	0.41	0.41	0.41	0.40
Meat and bone meal		5.30	5.31	5.32	4.89	4.89	4.90	4.24	4.24	4.25
Coccidiostat		0.05	0.05	0.05	0.05	0.05	0.05	0.05	0.05	0.05
Min-Vit Premix^6^		0.20	0.20	0.20	0.20	0.20	0.20	0.15	0.15	0.15
NaCl		0.41	0.41	0.41	0.34	0.34	0.34	0.36	0.36	0.36
L-Lysine HCl 78.8%		0.27	0.29	0.29	0.17	0.14	0.16	0.00	0.01	0.00
DL-Methionine 99%		0.28	0.31	0.31	0.25	0.22	0.25	0.16	0.19	0.19
L-Threonine 98%		0.06	0.08	0.08	0.03	0.01	0.03	-	-	-
Choline Chloride		0.05	0.05	0.05	0.05	0.05	0.05	0.05	0.05	0.05
Total		100	100	100	100	100	100	100	100	100
Calculated nutrients (%)										
Protein		22.74	21.84	21.88	20.94	21.83	20.97	21.30	20.38	20.41
Calcium		0.90	0.90	0.90	0.84	0.84	0.84	0.76	0.76	0.76
Total Phosphorus		0.67	0.66	0.65	0.64	0.65	0.63	0.61	0.61	0.60
Available Phosphorus		0.45	0.45	0.45	0.42	0.42	0.42	0.38	0.38	0.38
Sodium		0.22	0.22	0.22	0.19	0.19	0.19	0.19	0.19	0.19
TME^5^ kcal/kg		3285	3285	3285	3358	3358	3358	3430	3430	3430
Total amino acids	Lysine	1.32	1.32	1.32	1.19	1.19	1.19	1.061	1.05	1.05
	Methionine	0.62	0.64	0.64	0.56	0.55	0.56	0.48	0.50	0.50
	Methionine + Cysteine	0.98	0.98	0.98	0.89	0.89	0.89	0.82	0.82	0.82
	Tryptophan	0.26	0.26	0.26	0.25	0.25	0.25	0.25	0.25	0.25
	Threonine	0.86	0.86	0.86	0.78	0.78	0.78	0.77	0.74	0.75
	Arginine	1.45	1.41	1.42	1.37	1.40	1.37	1.38	1.34	1.35
	Valine	1.07	1.04	1.04	0.99	1.03	1.00	1.01	0.97	0.98

^1^IND: Event IND-ØØ412-7;^2^ Cadenza: non-GM counterpart;^3^ BioINTA: Commercial reference variety BioINTA 3005;^4^ CP: Crude protein;^5^TME: True metabolizable energy.^6^ Min-Vit Premix: Minerals and vitamins premix: vitamin A, 5,300,000 IU; vitamin D3, 1,560,000 IU; vitamin E, 15 g; vitamin K3, 1.7 g; thiamine, 1.0 g; riboflavin, 4.0 g; pyridoxine,2.0 g; vitamin B12, 0.01 g; niacin, 26.0 g; pantothenic acid, 6.5 g; folic acid, 0.6 g; biotin, 0.05 g; choline chloride, 60.0 g; Cu, 4.0 g; Fe, 30.0 g; Mn, 50.0 g; I, 0.1 g; Zn, 40.0 g; Se, 0.14 g.

### Measurements

Chickens were examined twice daily for general health. Bodyweight (BW, g) and feed intake (FI, g) adjusted for mortality (determined weekly), and feed conversion ratio (FCR) were calculated (adjusted feed intake: body weight, g:g) by pen for the same time intervals. At the end of the trial (42 days of age), 15 birds per treatment (taken from a single pen with an average BW similar to the whole set) were selected for analysis of meat yield (carcass yield, breast yield, and abdominal fat pad content). At the end of the study, all birds were sacrificed after electrical stunning by severing the jugular vein and carotid artery. Before composting, total bleeding and the death of the birds were confirmed. All chickens that died as well as those sacrificed at the end of the trial were composted following the recommendations of the National Service of Agri-food Health and Quality (SENASA, Argentina).

### Statistical analyzes

Data were analyzed by a two-way ANOVA using the General Linear Model procedure of InfoStat version 2017,^24^ and one-way ANOVA was used for carcass yield, breast yield, and abdominal fat pad content. Duncan’s multiple range test was used to determine differences between means at a significant level of 0.05.

## Results and discussion

As inclusion criteria, materials were tested for the presence of mycotoxins. No detectable levels were found for aflatoxins (B1, B2, G1, G2), ochratoxin, citrinin, zearalenone, T2 toxin, fusarenone, nivalenol, and diacetoxyscirpenol. The exception was deoxynivalenol (DON), often found in wheat in Argentina^25^ whose levels were 600 and 1000 ppb for the IND-ØØ412-7 event and its non-GM counterpart Cadenza, respectively. However, these values were below the tolerance levels for broiler chicken feed.^26^

The compositional equivalence between wheat event IND-ØØ412-7 and Cadenza has been previously reported.^27^ Before preparing the diets included in this study, the similarity of the composition of the three materials used in the study was examined by near-infrared spectroscopy (NIR, Table 2). As expected, no major differences in the main nutritional components were observed enabling the process to proceed to diets preparation.

**Table 2.** Grain composition as analyzed by NIR (% as is)

Component	IND-ØØ412-7	Cadenza	BioINTA 3005
Dry matter	88.78	89.46	88.95
Crude Protein	13.56	11.19	11.25
Ether Extract	2.1	2.2	2.1
Crude Fiber	2.9	3.1	2.9
ADF^1^	3.5	3.6	3.4
NDF^2^	13.0	13.0	12.3
Ash	2.0	2.1	2.0
Carbohydrates	2.2	2.5	2.4
Total Phosphorus	0.29	0.28	0.26
Methionine (Met)	0.205	0.176	0.173
Cysteine (Cys)	0.292	0.254	0.250
Met+Cys	0.495	0.426	0.422
Lysine	0.369	0.327	0.322
Threonine	0.379	0.323	0.322
Tryptophan	0.152	0.137	0.135
Arginine	0.635	0.540	0.539
Isoleucine	0.449	0.370	0.371
Leucine	0.895	0.746	0.747
Valine	0.553	0.466	0.468
Histidine	0.307	0.257	0.260
Phenylalanine	0.620	0.501	0.510

IND-ØØ412-7: Event tested; Cadenza: non-GM counterpart, BioINTA 3005: Commercial reference variety;^1^ Acid Detergent Fiber;^2^ Neutral Detergent Fiber

Values of Gross Energy (GE), True Metabolizable Energy (TME), and TME/GE ratio of the three materials are shown in Table 3. Similar values for GE and TME were found between wheat event IND-ØØ412-7 and its non-GM counterpart Cadenza. Both these parameters were statistically lower for the commercial variety BioINTA 3005 when compared to IND-ØØ412-7 and Cadenza (Table 3). This difference is likely related to the genetic background, shared by the transgenic material and its parental line, but different for BioINTA 3005. Crop variety is known to be among the numerous factors affecting the energy content and consequently the nutritional value of crop-derived feed. It is also affected by location, post-harvest treatment, and handling.^28^ Given that location, treatment, and handling were the same for the three materials included in the study, the lower GE and TME values for BioINTA 3005 can be attributed to a variety effect. However, all three genotypes show the same TME/GE ratio (Table 3).

**Table 3.** Energy levels of wheat genotypes

	IND-ØØ412-7	Cadenza	BioINTA 3005
Gross Energy (GE)	3891 ± 14^a^	3871 ± 16^a^	3662 ± 2^b^
True Metabolizable energy (TME)	3345 ± 42^a^	3329 ± 36^a^	3162 ± 39^b^
TME/GE	86.0 ± 1.1	86.0 ± 0.9	86.4 ± 1.1

GE and TME are expressed in kcal/kg as is; TME/GE is expressed as a percentage. Values represent mean ± SD; IND-ØØ412-7: Event tested; Cadenza: non-GM counterpart, BioINTA 3005: Commercial reference variety. Different letters within a row indicate a significant difference (*p* < 0.05).

Cumulative feed intake (FI), and feed conversion ratio (FCR) were similar for wheat event IND-ØØ412-7 and Cadenza (Table 4), showing the nutritional equivalence of these parameters for the transgenic event to its non-GM counterpart. Significantly higher BW values were observed in chickens fed with the BioINTA 3005 commercial reference when compared to the other two materials. Again, this difference can be attributed to the genetic background effect. These sporadic differences were observed at early stages along the trial and vanished at the last growing phase (Table 4). These sporadic differences could arise from digestibility variations associated with different contributions of diet ingredients, as diets composition was calculated to equalize TME and amino acids levels, which may have required slight differences in ingredient levels.

**Table 4.** Broiler performance

Parameter		Cumulative Feed Intake^1^ (FI, g)			Body Weight (BW, g)			Feed Conversion Ratio (FI/BW, g:g)		
Diet	Day	IND^2^	Cadenza^3^	BioINTA^4^	IND^2^	Cadenza^3^	BioINTA^4^	IND^2^	Cadenza^3^	BioINTA^4^
Starter	7	138 ± 1	139 ± 1	141 ± 5	172 ± 3	170 ± 3	173 ± 2	0.80 ± 0.01	0.82 ± 0.01	0.81 ± 0.02
	14	590 ± 11	589 ± 11	597 ± 17	496 ± 4^a^	495 ± 7^a^	511 ± 6^b^	1.19 ± 0.03	1.19 ± 0.03	1.17 ± 0.03
Grower	21	1391 ± 45	1391 ± 27	1419 ± 25	1049 ± 28^a^	1039 ± 27^a^	1084 ± 29^b^	1.33 ± 0.05	1.34 ± 0.02	1.31 ± 0.04
	28	2407 ± 95	2390 ± 48	2426 ± 46	1685 ± 49^ab^	1660 ± 36^a^	1720 ± 33^b^	1.43 ± 0.04	1.44 ± 0.02	1.41 ± 0.03
Finisher	35	3819 ± 111	3795 ± 67	3822 ± 79	2476 ± 29	2472 ± 13	2504 ± 31	1.54 ± 0.04	1.54 ± 0.03	1.53 ± 0.02
	42	5101 ± 154	5060 ± 100	5014 ± 100	3169 ± 48	3170 ± 27	3155 ± 73	1.61 ± 0.02	1.60 ± 0.03	1.59 ± 0.02

^1^Measured weekly;^2^ IND: Event IND-ØØ412-7;^3^ Cadenza: non-GM counterpart;^4^ BioINTA: Commercial reference variety BioINTA 3005; Values represent mean ± SD; Different letters within a row and parameter indicate statistical significance (*p* < 0.05).

The identical nutritional performance of wheat event IND-ØØ412-7, the non-GM near-isogenic counterpart, and the commercial variety, as measured by the efficiency of feed utilization, was confirmed by comparing the time (days) to reach a commercial slaughter weight of 2.8 kg.^29^ The time span for IND-ØØ412-7 was 37.8 ± 0.9 days, and similar values were obtained for the conventional wheat varieties tested in parallel (37.8 ± 0.3 and 37.8 ± 1.4 days for Cadenza and BioINTA 3005, respectively).

No differences between IND-ØØ412-7 and Cadenza were found in the meat yield parameters analyzed (Table 5), although carcass yield was slightly but significantly lower for these two genotypes compared to the commercial reference BioINTA 3005. Again, as diets were formulated on both, identical TME and amino-acids levels basis, these differences may reflect different contributions of diet ingredients and are unlikely to have biological significance as they are not found in the energy- and feed-efficiency- related endpoints.

**Table 5.** Meat yield characteristics

Parameter	Units	IND-ØØ412-7	Cadenza	BioINTA 3005
Carcass Yield	%	74.3 ± 1.4 ^a^	74.4 ± 1.2 ^a^	75.6 ± 1.3 ^b^
Breast Yield	g	870 ± 67	870 ± 72	876 ± 66
	%	28.2 ± 1.5	28.5 ± 1.1	29.1 ± 1.6
Abdominal Fat Pat Content	g	36.7 ± 11.9	44.8 ± 13.3	44.9 ± 13.9
	%	1.19 ± 0.38	1.45 ± 0.38	1.50 ± 0.45

Values represent mean ± SD; %: percentage of body weight. IND-ØØ412-7: Event tested; Cadenza: non-GM counterpart, BioINTA 3005: Commercial reference variety; Different letters within a row indicate a significant difference (*p* < 0.05).

Overall, no significant differences were found in the growth performance and feed conversion efficiency between chickens fed wheat event IND-ØØ412-7 and those fed the non-GM counterpart Cadenza.

We can therefore conclude that when used in balanced broiler diets, wheat event IND-ØØ412-7 is nutritionally equivalent to non-GM wheat.
